# Cap-assisted endoscopy increases ampulla of Vater visualization in high-risk patients

**DOI:** 10.1186/s12876-020-01361-5

**Published:** 2020-07-09

**Authors:** Leonardo Correa Silva, Rondinelle Martins Arruda, Paula Fortuci Resende Botelho, Leonardo Nogueira Taveira, Kelly Menezio Giardina, Marco Antonio de Oliveira, Julia Dias, Cleyton Zanardo Oliveira, Gilberto Fava, Denise Peixoto Guimarães

**Affiliations:** 1 Department of Endoscopy, Barretos Cancer Hospital, Antenor Duarte Villela, 1331, Zip Code: 14784 400, Barretos, São Paulo, Brazil; 2 Department of Biostatistics, Barretos Cancer Hospital, Barretos, SP Brazil; 3 Molecular Oncology Research Center, Barretos Cancer Hospital, Barretos, Brazil

**Keywords:** Cap-assisted endoscopy, Forward-viewing endoscopy, Ampulla of Vater, Ampullary carcinoma, Ampullary adenoma

## Abstract

**Background:**

Periampullary adenocarcinoma is a major clinical problem in high-risk patients including FAP population. A recent modification for visualizing the ampulla of Vater (AV) involves attaching a cap to the tip of the forward-viewing endoscope. Our aim was to compare the rates of complete visualization of AV using this cap-assisted endoscopy (CAE) approach to standard forward-viewing endoscopy (FVE). We also determined: (i) the rates of complications and additional sedation; (ii) the mean time required for duodenal examination; and (iii) the reproducibility among endoscopists performing this procedure.

**Methods:**

We performed esophagogastroduodenoscopy for AV visualization in 102 > 18 years old using FVE followed by CAE. Video recordings were blinded and randomly selected for independent expert endoscopic evaluation.

**Results:**

The complete visualization rate for AV was higher in CAE (97.0%) compared to FVE (51.0%) (*p* <  0.001). The additional doses of fentanyl, midazolam, and propofol required for CAE were 0.05, 1.9 and 36.3 mg. in 0.9, 24.5, and 77.5% patients, respectively. The mean time of duodenal examination for AV visualization was lower on CAE compared to FVE (1.41 vs. 1.95 min, *p* <  0.001). Scopolamine was used in 34 FVE and 24 CAE, with no association to AV complete visualization rates (*p* = 0.30 and *p* = 0.14). Three more ampullary adenomas were detected using CAE compared to FVE. Cap displacement occurred in one patient, and there was no observed adverse effect of the additional sedatives used. Kappa values for agreement between endoscopists ranged from 0.60 to 0.85.

**Conclusions:**

CAE is feasible, reproducible and safe, with a higher success rate for complete visualization compared to FVE.

Trial registration: ClinicalTrials.gov, NCT02867826, 16 August 2016.

## Background

Ampullary carcinoma is a rare disease with an incidence of 3.8 cases / 1,000,000 in men and 2.7 / 1,000,000 in women [1], and it is responsible for 20% of tumors that obstruct the common bile duct [2]. The incidence of this carcinoma is significantly higher in the context of hereditary syndromes, such as familial adenomatous polyposis (FAP) and hereditary nonpolyposis colorectal cancer (HNPCC), with an increased risk of 200-300X compared to the general population [3].

Complete visualization of the ampulla of Vater (AV) during screening upper GI endoscopy is important as it allows detection of early ampullary neoplasia [4, 5]. Limitations of the conventional esophagogastroduodenoscopy (EGD) using forward-viewing endoscope in visualizing the AV are mainly due to the tangential position of the AV, presence of anatomical variants in certain patients such as overlying duodenal folds or periampullary diverticulum and inability to achieve complete straightening in certain cases.

European and American guidelines recommend using a side-viewing duodenoscope as the gold standard for surveillance of patients at high-risk or with a suspected ampullary neoplasia [4, 6–8]. Unfortunately, this type of duodenoscope is not always available in an outpatient setting as it is expensive, requiring a high level of handling expertise, and it is less tolerated owing to the larger diameter, which requires deeper sedation.

A number of studies have been conducted to improve the rate of complete AV visualization using the standard forward-viewing endoscope One technique to improve visualization rate is to straighten the scope by withdrawing it when the tip of the endoscope reach the proximal level of the descending duodenum. Hew et al. showed that this technique increased the rate of detection by 21.3% (33.4% vs. 54.7%), compared to the normal method of advancing the scope into the descending duodenum [9]. In addition to the straightening maneuver, the use of a transparent cap fitted to the tip of the forward-viewing endoscope (Cap-assisted endoscopy) has been suggested as a way to improve AV visualization [10, 11]. The use of a cap fitted to the end of the colonoscope has also been found to aid the inspection of the blind areas of the colonic mucosa behind folds, reducing polyp miss rate and decreasing the cecal intubation time [12–14]. In selected patients in which AV had been missed by forward-viewing endoscopy, Choi et al. showed that a complete AV visualization rate of up to 91.3% can be achieved in this group of patients when re-examined with CAE [10].

There are presently few studies regarding the efficacy and safety of CAE in patients referred for AV visualization. Our primary aim was therefore to ascertain the efficacy of CAE for complete visualization of the AV in patients indicated for examination. Our secondary aims were to assess the complication rates of CAE, the mean time required for duodenum examination to visualize AV and any additional doses of sedation for this procedure compared to FVE.

## Methods

### Study design

This was a prospective and comparative study conducted at Barretos Cancer Hospital, Brazil, between August 2016 and January 2018. The study was approved by the local research committee (Research Ethical Committee, n° 1151/2016) and registered at ClinicalTrials.gov (NCT02867826, 16/08/2016). All research was performed in accordance with relevant guidelines and written informed consent was obtained from all patients.

### Patients

The inclusion criteria were age > 18 years old and those scheduled for EGD with the intention of examining the AV. The exclusion criteria were previous upper gastrointestinal tract surgery, previous endoscopic ampullectomy and any contraindication for elective EGD or patients who refused to provide an informed consent.

### Endoscopic procedure

All patients underwent a FVE followed by aCAE on the same day, in an outpatient setting. The procedure was performed by a GI endoscopy resident under the supervision of a senior endoscopist. Three residents participated, and each procedure was supervised by 1 of 5 experienced endoscopists (L.N.T., K.M.G., J.C.V.D., G.F. and D.P.G.). Olympus gastroscope (GIF-H180J; Olympus Optical Co., Ltd., Brazil) and duodenoscope (TJF-160VR; Olympus Optical Co., Ltd., Brazil) were used in this study. A transparent soft cap with an outer diameter of 12.4 mm and length from the distal end of endoscope of 4 mm (Disposable Distal Attachment, Model D-201–11,804; Olympus Medical Systems Inc.) was used for the cap-assisted upper GI endoscopy.

The procedure was performed with patients in the left lateral decubitus position under moderate sedation, using intravenously administered fentanyl (50 mcg), midazolam (1 mg/ml saline solution, initial dose of 3 mg), and propofol (initial dose of 30 mg) [15]. Additional doses of midazolan (maximum dosage of 5 mg) and additional bolus of 20 mg propofol were used as deemed needed to maintain the sedation level. Supplemental oxygen was administrated to all patients during the endoscopic procedure via nasal cannulae. Scopolamine was used at the discretion of the endoscopist. Pulse oximetry, heart rate and blood pressure were monitored.

AV biopsies and duodenal polypectomy were taken, if indicated. The stage of duodenal polyposis was graded according to Spigelman classification [16]. The ENDOX® system software (Tesi, Italy) was used for acquisition and archiving images and movies in all endoscopic procedures. In both procedures, AV visualization was video-recorded. The time for the AV visualization was recorded from the moment we accessed the second part of the duodenum up to the AV visualization. Once we determined whether the AV was complete or partially visualized, the counting time stopped.

### Video-based assessments

Videos were given a random number identification within the FVE and CAE groups through a code created by the Department of Biostatistics of the Barretos Cancer Hospital. Video recording of the same procedure was assessed by two blinded senior endoscopists (each faculty members) for identification data and for evaluation of the results of the EGD. AV visualization was described.

### AV visualization description

**Fig. 1:**
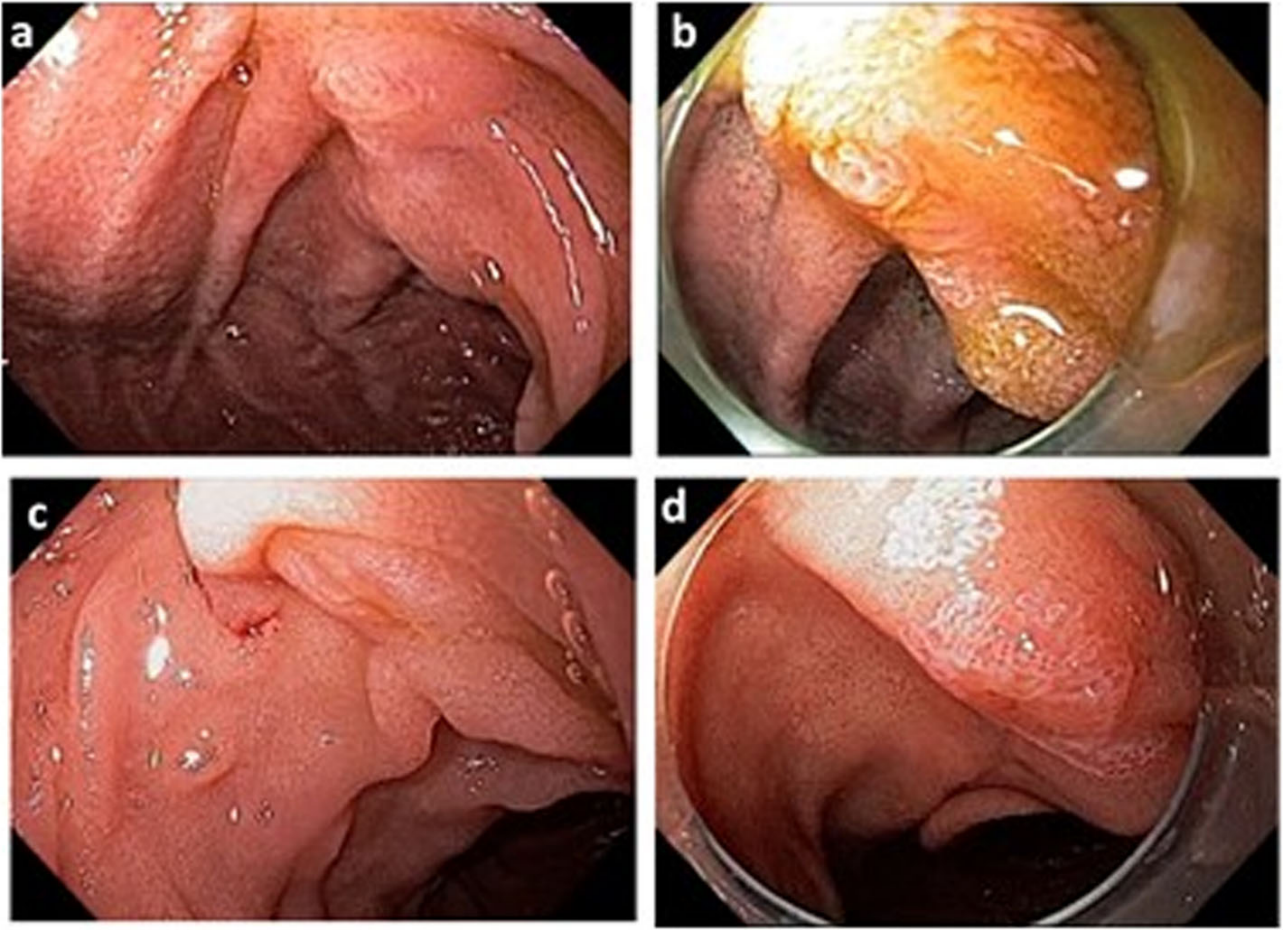
Representative cases of ampulla of Vater visualization on forward-viewing endoscopy and cap-assisted endoscopy. AV completely visualized with FVE (**a**) and CAE (**b**). AV partially visualized with FVE (**c**) followed by CAE with completely visualization and as ampullary adenoma (**d**)

The AV visualization was categorized during the endoscopy (by the resident under the supervision of a senior endoscopist) and during the review of the video recording (by the blinded senior endoscopists) as follow: i) completely visualized (visualization of all structures of the AV: the frenulum, the hood, the infundibulum, and the orifice of the major papilla); ii) partially visualized (visualization of only part of the AV structures); or iii) not visualized (Fig. 1).

### Outcomes

Primary: complete AV visualization.

Secondary: rate of complications, additional sedation dose and time of AV examination and reproducibility of performing CAE procedure among the endoscopy experts.

### Sample size and statistical analysis

Based on the data from Choi et al. [10], we can assume a 15% difference for complete AV visualization in the comparison between FVE and CAE and with an 80 and 90% test power, the sample size estimated was 91 and 121 patients, respectively. All data was collected using a study-specific REDCap database [17].

Descriptive statistics were expressed in number, percentage, mean and standard deviation. For the interobserver agreement, kappa statistics were calculated. For comparison of AV visualization, the McNemar test was used. The chi-square or Fisher exact test were used to study association between the use of Scopolamine and AV visualization. The Student t-test for normally distributed data or Mann Whitney (U) test for not normally distributed data, were used for comparison of the time of AV examination between FVE and CAE. All *p*-values were considered significant at the 0.05 level. All statistical analyses were performed using SPSS statistical software version 21.

## Results

### Population description

**Fig. 2:**
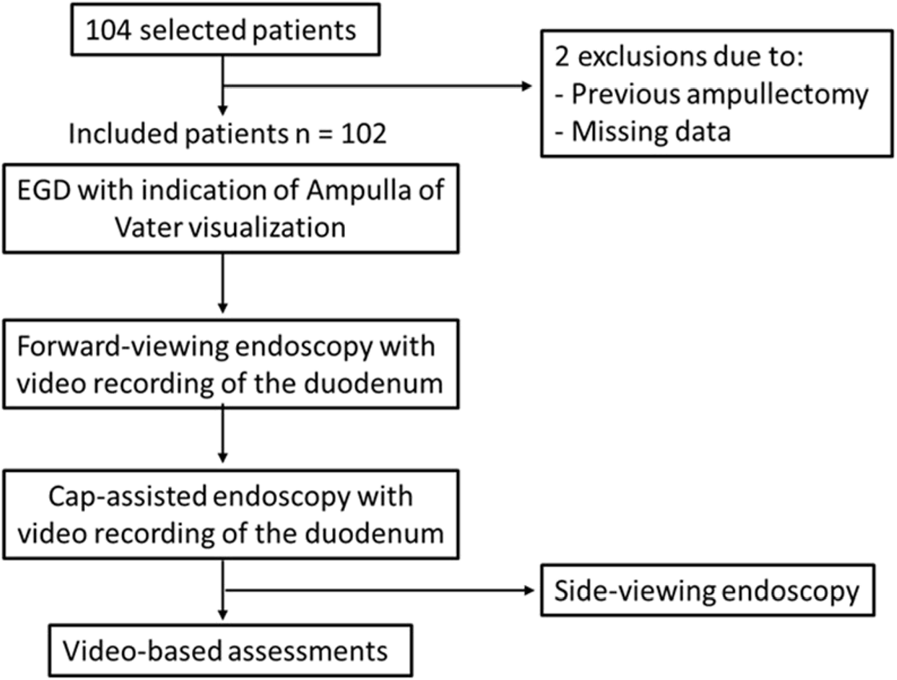
Flow diagram of patients undergoing Forward-viewing endoscopy (FVE) and Cap-assisted endoscopy (CAE) selected for the study

A sample of 121 and 91 patients would provide the trial with 90 and 80% power, respectively, to obtain a significant difference with *p* <  0.05 on a two-sided test. We decided to stop patient inclusion when the minimum sample size providing the trial with 80% power was achieved, upon approval by the local research committee. One hundred and four consecutive patients referred for EGD with an indication of a need for AV visualization were included in this study. Two patients were excluded, one due to the identification of post ampullectomy scar during the EGD and one because of inadequate data. A total of 102 patients completed the study protocol (Fig. 2). Clinical and demographic characteristics are described in Table 1. The patient mean age was 41.7 years (+ 14.4), and 60.8% were female. The main indication for EGD was surveillance in FAP patients (84.3%). Among these patients, 72.0% had undergone total colectomy and in 55 (64.0%) patients the severity of duodenal disease classified as Spigelman stages II, III and IV (Table 1).

**Table 1:** Demographic and baseline features of patients

Data	***n*** = 102
Female, n (%)	62 (60.8)
Age, years	41.7 + 14.4
BMI, kg/m^2^	25.3 + 5.6
Indication for EGD, n (%)	
FAP	86 (84.3)
Ampullary neoplasia	10 (9.8)
Pancreatic neoplasia	6 (5.9)
**FAP patients**	***n*** **= 86**
Total colectomy, n (%)	62 (72.0)
Spigelman stage, no (%)	
0	21 (24.4)
I	10 (11.6)
II	42 (48.8)
III	12 (14.0)
IV	1 (1.2)

*BMI* Body Mass Index, *EGD* esophagogastroduodenoscopy, *FAP* Familial Adenomatous Polyposis, *SD* standard deviation

Values given as mean + SD or n (%)

### Ampulla of Vater visualization rates with CAE and FVE

In the FVE group, the AV was completely visualized in 52 (51.0%) patients, partially visualized in 37 (36.3%) and not visualized in 13 (12.7%) patients. In the CAE group, the AV was completely visualized in 99 (97.0%) patients, partially in 1 (1.0%) and not visualized in 2 (2.0%) patients (*p* <  0.001, Table 2).

**Table 2:** Comparison of ampulla of Vater visualization between FVE and CAE

Ampulla of Vater visualization	FVE	CAE	***P*** value
Completely visualized	52 (51.0%)	99 (97.0%)	< 0.001
Partially or not visualized	50 (49.0%)	3^a^ (3.0%)	< 0.001

*FVE* forward-viewing endoscopy, *CAE* cap-assisted endoscopy

^a^side-viewing endoscopy were performed

The mean time of AV visualization in FVE and CAE were calculated. This comparison showed that the mean time was lower on CAE than on FVE (1.41 + 0.95 vs. 1.95 + 1.52 min, *p* < 0.001).

A side-viewing endoscopy was required in three patients in whom the AV was not fully visualized even with CAE.

### Use of sedatives and scopolamine

Additional doses of 0.05 ± 0 mg of fentanyl were administrated in 0.9% of patients, 1.9 ± 0.5 mg of midazolam in 24.5% of patients and 36.3 ± 26.5 mg of propofol in 77.4% of patients for maintaining appropriate levels of sedation during CAE (Table 3). Scopolamine was used at the discretion of the endoscopist to reduce intestinal peristalsis in 33.3% of FVE and 23.5% of CAE. Thus, an association analysis was performed to verify the influence of scopolamine on the AV visualization rate. This analysis showed that visualization rates in FVE and CAE were not significantly different between examinations performed with or without scopolamine. (*p* = 0.30 and *p* = 0.14, respectively) (Table 4).

**Table 3:** Use of sedatives during the endoscopy

	FVE		CAE	
	n (%)	Doses, mg	n (%)	Doses, mg
Fentanyl	102 (100.0)	0.05 + 0	1 (0.9)	0.05 + 0
Midazolam	102 (100.0)	3.8 + 1.2	25 (24.5)	1.9 + 0.5
Propofol	102 (100.0)	51 + 25.5	79 (77.5)	36.3 + 26.5

*FVE* forward-viewing endoscopy, *CAE* cap-assisted endoscopy

Values given as mean + SD or n(%). SD, standard deviation

**Table 4:** The influence of the use of scopolamine on the AV visualization rate during the FVE and CAE

Use of scopolamine	FVE			CAE		
	AV visualization (%).		*P* value	AV visualization (%).		*P* value
	Completely visualized	Partially/not visualized		Completely Visualized	Partially/not visualized	
Yes	20 (38.5)	14 (28.0)	0.30	22 (22.2)	2 (66.7)	0.14
No	32 (61.5)	36 (72.0)		77 (77.8)	1 (33.3)	

*FVE* forward-viewing endoscopy, *CAE* cap-assisted endoscopy

Values given as no

### Endoscopic findings and complications

Suspicious areas were detected in 36 (35.2%) patients in FVE and 46 (45.0%) in CAE (Table 5). All suspicious lesions seen in FVE were also seen by CAE. Histological analysis confirmed adenomas in 26 (25.4%) patients in the FVE examination and 29 (28.4%) adenomas by CAE (Table 5). Among adenomas, 26 (89.6%) were tubular with low-grade dysplasia, and three (10.4%) were tubulovillous adenoma with high-grade dysplasia. Adenocarcinoma was detected in one patient, by both FVE and CAE. For the three patients who required a side-viewing endoscopy for adequate AV evaluation, no suspicious lesions were seen.

**Table 5:** Description of endoscopic findings in both FVE and CAE

***n*** = 102	FVE	CAE
Suspicious lesion	36 (35.2)	46 (45.0)
*Subepithelial lesions*	0 (0.0%)	1 (1.0)
Histological analysis		
Negative for neoplasia	9 (8.8)	15 (14.7)
Adenocarcinoma	1 (1.0)	1 (1.0)
Adenoma	26 (25.4)	29 (28.4)

*FVE* forward-viewing endoscopy, *CAE* cap-assisted endoscopy, *NE* not evaluated, *SD* standard deviation

Values given as mean + SD or n (%)

Three complications were reported in the study. One mild trauma of the duodenal mucosa by both FVE and CAE in the same patient. Cap displacement occurred in one patient, which was promptly recovered using biopsy forceps. There were no reports of adverse effects related to additional doses of sedatives that were used.

### Reproducibility of the exam

In addition, we evaluated the agreement of AV visualization between the examiner and each of the other two observers, as well as interobserver agreement. Kappa values for agreement between the observers ranged from 0.60 to 0.85. This level of reproducibility was considered moderate to excellent.

## Discussion

The success rate of AV visualization was recently improved by fitting a transparent cap to the tip of a forward viewing endoscope as an alternative approach to conventional side-viewing endoscopy. We conducted a prospective study to compare CAE with FVE in 102 patients referred for AV visualization. In 97% of patients, we found that CAE could completely visualize AV with low rates of complications, compared to only 51% using FVE. Our findings also demonstrated that CAE can overcome some of the limitations of FVE for AV inspection, suggesting this modification to the endoscope is a safer and more accessible surveillance option for detecting AV neoplasia in high-risk patients.

Since our institution follows a large number of families with hereditary cancer associated syndromes, 80% of our study population referred for AV visualization were FAP patients. This group of patients has a high prevalence of duodenal or AV neoplastic diseases or anatomical variations due to postoperative adhesions in cases of previous colectomy. For the upper and lower GI surveillance endoscopy, these patients are usually referred to an outpatient endoscopy clinic in our Institution where a side viewing endoscope is not always available. Before this study, patients in whom the AV was not completely visualized by FVE were sent to the advanced endoscopy center in our Institution. The results of this study show that CAE had a high rate of success at AV visualization so that we changed our routine for all patients with hereditary cancer syndromes to perform CAE exclusively for upper GI endoscopy. Importantly, the duodenal and gastric surveillance for neoplasia was also performed by CAE, and no difficulties or limitations in the field of view were encountered when the cap was used. These findings suggest that routine endoscopy of the AV can be done using the cap-assisted procedure without prejudicing examinations in the other regions of interest.

The rate of complete AV visualization with CAE was similar to the finding from two other previous studies that both included examinations of healthy patients [10, 11] and one study that just involved FAP patients [18] (91.3, 97, and 95%, respectively). In contrast to these comparisons, our rate of complete AV visualization with FVE was quite different to that of Abdelhafez et al. [11] (51% vs. 80.8%) and of Choi [10] (51% vs. 24%). However, it was similar to the rate of 54.7% found by Hew et al. [9], which in our view, represent a reasonable rate for FVE.

In our study, both the FVE and CAE procedures were only performed by endoscopy residents, who had no previous experience with ERCP using the side-viewing endoscopes. We obtained the similar rates of complete AV visualization as previous studies where the exams were performed by experienced endoscopists with ERCP (91.3%) [10] and endoscopist who did not routinely perform ERCPs (95%) [18] suggesting that CAE is a simple procedure, with faster learning curve than ERCP and can be generalized to conventional endoscopists. For three patients, AV was partially or failed to be visualized even with CAE. In these patients, an additional examination using a side-viewing endoscope was performed to detect AV. For one of these patients, the difficulty in assessing the AV by both FVE and CAE was likely due to the presence of a tiny AV and the lack of stability of the forward-viewing endoscope position at the second part of the duodenum. In another patient, there was also instability, but this was probably attributed to the adhesions post total colectomy since AV had already been fully evaluated with FVE before the surgical procedure.

Interobserver agreement was ascertained through the evaluation of the duodenal videos and was considered moderate to excellent (kappa range of 0.60 to 0.85). We found a slightly lower concordance than that described by Abdelhafez et al. (kappa range 0.93 to 0.95), in which the evaluation was made through endoscopic photos [11]. This difference can be attributed to the fact that video-recordings are more representative of the clinical situation than static images captured during the endoscopic procedure. Another of our observations was that the mean time of AV visualization was significantly faster in the CAE compared to FVE, 1.41 vs. 1.95 min, respectively. This result suggests that the AV is more readily localized by CAE. Nevertheless, it is worth mentioning that observing AV first with FVE may facilitate AV visualization with CAE due to memory bias and therefore, it could impact on the lower time of CAE.

Recently, two non-inferiority randomized trials compared AV viewing rates between CAE and side-viewing endoscopy and showed different results. Abdelhafez et al. obtained a complete AV viewing rate of 95% in the CAE group vs. 97% in the duodenoscopy group, not exceeding the 8% margin of difference established in their study [19]. On the other hand, Shi et al. fails to demonstrate the non-inferiority of the CAE [20]. The AV visualization was achieved in 68.2% in the CAE group and in 86.0% in duodenoscopy group [20]. Although we did not compare CAE and duodenoscopy, our AV visualization rate in the CAE are similar to Abdelhafez et al. (97% vs 95%) [19]. The lower AV viewing rate found by Xin et al. can be justified by the characteristic of the population included in the study, all patients with indications for ERCP, with a higher number of AV pathologies and abnormalities, compared to healthy patients included in the study by Abdelhafez [19]. Although our population is composed of patients at high risk for AV neoplasia, in most cases, AV does not present any abnormality, resembling the study population of Abdelhafez et al. [19].

Collectively our study suggests that there are two options for side-viewing endoscopy in high-risk patients: Conventional EGD (FVE), followed by CAE for ampullary and duodenal evaluation, or an exclusive cap-assisted esophagogastroduodenoscopy. Nevertheless, this study has some limitations that should be mentioned. Firstly, we did not perform a comparative study between CAE and side-viewing endoscopy, the gold standard method. Secondly, this study was not randomized, and the endoscopists were not blinded due to the visibility of the cap in some examinations, leading to the potential for bias in favour of CAE. Subjective interpretations could only be partially minimized by the random video evaluation. Furthermore, the cost-effectiveness of CAE was not addressed in the present study. However, in our opinion, examining with the cap is likely to be less costly, due to the lower dose of sedation than SVE, and mainly because there is no need for the duodenoscope, which prevents the cost and risks of its reprocessing.

## Conclusion

Cap-assisted endoscopy is feasible and safe, with higher success for complete visualization of AV, compared to forward-viewing endoscopy, which can be generalized to conventional endoscopists. Finally, the reproducibility between endoscopists was moderate to excellent.

## Data Availability

The datasets generated and analysed during this study are included available from the corresponding author on reasonable request.

## Abbreviations

FAP: Familial adenomatous polyposis
AV: Ampulla of Vater
CAE: Cap-assisted endoscopy
FVE: Standard forward-viewing endoscopy
HNPCC: Hereditary nonpolyposis colorectal cancer
GI: Gastrointestinal
EGD: Esophagogastroduodenoscopy
